# Endothelial S1pr1 regulates pressure overload‐induced cardiac remodelling through AKT‐eNOS pathway

**DOI:** 10.1111/jcmm.14900

**Published:** 2019-12-19

**Authors:** Xiuxiang Liu, Jinjin Wu, Chenying Zhu, Jie Liu, Xiaoli Chen, Tao Zhuang, Yashu Kuang, Yanfang Wang, Hao Hu, Ping Yu, Huimin Fan, Yuzhen Zhang, Zhongmin Liu, Lin Zhang

**Affiliations:** ^1^ Key Laboratory of Arrhythmias of the Ministry of Education of China Research Center for Translational Medicine Shanghai East Hospital Tongji University School of Medicine Shanghai China; ^2^ Cardiovascular Department Shanghai Children's Medical Center Shanghai Jiaotong University Shanghai China; ^3^ Heart Failure Institute Shanghai East Hospital Tongji University School of Medicine Shanghai China; ^4^ Department of Cardiology Shanghai East Hospital Tongji University School of Medicine Shanghai China

**Keywords:** cardiac fibrosis, cardiac hypertrophy, heart failure, pathological cardiac remodelling, sphingosine 1‐phosphate receptor 1, vascular endothelial cells

## Abstract

Cardiac vascular microenvironment is crucial for cardiac remodelling during the process of heart failure. Sphingosine 1‐phosphate (S1P) tightly regulates vascular homeostasis via its receptor, S1pr1. We therefore hypothesize that endothelial S1pr1 might be involved in pathological cardiac remodelling. In this study, heart failure was induced by transverse aortic constriction (TAC) operation. S1pr1 expression is significantly increased in microvascular endothelial cells (ECs) of post‐TAC hearts. Endothelial‐specific deletion of *S1pr1* significantly aggravated cardiac dysfunction and deteriorated cardiac hypertrophy and fibrosis in myocardium. In vitro experiments demonstrated that S1P/S1pr1 praxis activated AKT/eNOS signalling pathway, leading to more production of nitric oxide (NO), which is an essential cardiac protective factor. Inhibition of AKT/eNOS pathway reversed the inhibitory effect of EC‐S1pr1‐overexpression on angiotensin II (AngII)‐induced cardiomyocyte (CM) hypertrophy, as well as on TGF‐β‐mediated cardiac fibroblast proliferation and transformation towards myofibroblasts. Finally, pharmacological activation of S1pr1 ameliorated TAC‐induced cardiac hypertrophy and fibrosis, leading to an improvement in cardiac function. Together, our results suggest that EC‐S1pr1 might prevent the development of pressure overload‐induced heart failure via AKT/eNOS pathway, and thus pharmacological activation of S1pr1 or EC‐targeting S1pr1‐AKT‐eNOS pathway could provide a future novel therapy to improve cardiac function during heart failure development.

## 1 INTRODUCTION

Heart failure is a leading cause of morbidity and mortality worldwide.^1^ Cardiac hypertrophy develops as a compensatory response of the heart towards haemodynamic overload, such as hypertension and valvular heart disease.^1^ Under sustained pressure overload or heart injury, the heart can no longer compensate for haemodynamic alterations and transits from adaptive to maladaptive remodelling, eventually progressing to ventricular dysfunction and heart failure.^2^ Cardiac fibrosis is an important histological character of cardiac remodelling during the development of heart failure, and the deposition of excessive extracellular matrix proteins within myocardium contributes to ventricular dilatation and contractile dysfunction.^3^

As one of the most abundant cell types in the heart, endothelial cells play an important role in the regulation of cardiac remodelling.^3^ On one hand, vascular endothelial cells construct the inner layer of blood vessels in a thin monolayer pattern, and their homeostasis maintains cardiac vascularization and perfusion during physiological and pathological processes. On the other hand, endothelial cells contribute to the microvessels and cardiac endothelial cells communicated with adjacent cardiomyocytes and fibroblasts by the secretion of bioactive molecule, such as nitric oxide (NO) synthesized by endothelial NOS (eNOS),^4^ and thus play an important role in the process of pathological cardiac remodelling, including cardiac hypertrophy and fibrosis.^3^, ^5^

It has been known that sphingosine 1‐phosphate receptor 1 (S1pr1) was highly expressed in vascular endothelial cells and played a vital role in endothelial functions.^6^, ^7^ Previous studies have shown that EC‐S1pr1 tightly controlled the integrity of endothelium by stabilization of VE‐cadherin at endothelial junctions.^8^ Loss of S1pr1 or pharmacological blockade of S1pr1 resulted in increased permeability of blood vessels.^6^ Deletion of *S1pr1* gene in mice caused embryonic death at E12.5‐14.5 due to a severe defect in vasculature development.^6^ Given that EC‐S1pr1 plays an important role in the control of vascular homeostasis and that cardiac ECs exert an active player in cardiac physiology and pathology, we hypothesized that EC‐S1pr1 might influence cardiac remodelling during pressure overload‐induced heart failure.

To address this issue, we generated tamoxifen inducible vascular EC‐specific *S1pr1* loss‐of‐function mice to explore the roles and mechanisms of EC‐S1pr1 in pathological cardiac remodelling in a left ventricular (LV) pressure overload mouse model induced by transverse aorta constriction (TAC). The results indicated that EC‐S1pr1 loss‐of‐function mice developed more severe cardiac fibrosis and hypertrophy after TAC operation, compared to *WT* control littermates. Further studies showed that S1P/S1pr1 praxis activated AKT/eNOS signalling pathways and enhanced the production of NO, which contributed to the protective effect of EC‐S1pr1 on cardiac hypertrophy and fibrosis. Furthermore, pharmacological activation of S1pr1 significantly ameliorated pressure overload‐induced cardiac hypertrophy and fibrosis, and therefore improved cardiac function in vivo, suggesting that EC‐S1pr1 signals as a potential target to therapy heart failure.

## 2 MATERIALS AND METHODS

### 2.1 Generation of vascular endothelial cell‐specific S1pr1 loss‐of‐function mouse model

The conditional *S1pr*1 knock‐out (*S1pr1^flox/flox^*) mice were obtained from Jax Mice (Stock number 019141). Cdh5 promoter‐driven Cre line (*Cdh5‐Cre^ERT2^*) was a kind gift from Professor Adams RH’s laboratory.^9^ The conditional *S1pr1* loss‐of‐function (*S1pr1^flox/flox^*) mice were crossed with tamoxifen inducible Cdh5 promoter‐driven Cre line (*Cdh5‐Cre^ERT2^*) to generate EC‐specific *S1pr1* loss‐of‐function mice, *S1pr1^fl/fl^;Cdh5‐Cre^ERT2^* (*S1pr1^ECKO^*). All animal procedures were performed in accordance with the Institutional Animal Care and Use of Laboratory Animals approved by the Tongji University Institutional Animal Care and Use Committee with license number TJLAC‐0129‐026.

### 2.2 Transverse aortic constriction induced pathological cardiac remodelling models

*S1pr1^ECKO^* mice and *S1pr1^wt/wt^;Cdh5‐Cre^ERT2^* (wild‐type) littermates of 10‐12 weeks age were used for pathological cardiac remodelling studies. Tamoxifen (100 mg/kg mice) was administrated every other day for a total of four times via intraperitoneal injection (ip) prior to the experiments to induce EC‐specific S1pr1 deletion. The TAC pressure‐overload model was accomplished by ligation of the transverse aorta between right innominate and left common carotid arteries against a blunted 27‐gauge needle with a 7‐0 suture. The needle was then gently removed. The sham procedure was identical except that the aorta was not ligated. Echocardiography was performed at 28 days after surgery.

### 2.3 Echocardiography analysis

Echocardiography was performed to evaluate the cardiac geometry, systolic and diastolic function as previously described.^3^ A Visual Sonics high‐resolution Vevo2100 ultrasound system (VisualSonics Inc.) with a 30‐MHz linear array ultrasound transducer (MS‐400; VisualSonics Inc.) was used. In brief, mice were anaesthetized with 2.0% isoflurane until the heart rate stabilized at 400‐500 beats per minute. Parasternal long‐axis images were acquired in B‐mode with the scan head in an appropriate position to identify the maximum LV length. In this view, the M‐mode cursor was positioned perpendicular to the maximum LV dimension in end‐diastole and systole, and M‐mode images were obtained for measuring wall thickness and chamber dimensions. Left ventricle (LV) ejection fraction (EF) and LV fractional shortening (LVFS) were calculated automatically.

### 2.4 Reagents

L‐NAME (Sigma Aldrich, #N5751), LY294002 (Selleck, #S1105), angiotensin II (AngII, Sigma Aldrich, #A9525), TGF‐β (Peprotech, #10‐21), DMSO (Sigma, #D2650), S1P (Cayman, #62570), SEW2871 (Cayman, #10006440‐5), Anti‐Wheat Germ Agglutinin‐alexa488 (Invitrogen, #W11262), Biotinylated‐isolectin B4 antibody (IB4, Vector Laboratories, #B‐1205), TRITC‐phalloidin (Yeasen, #40734ES75), DAPI (Sigma Aldrich, #D9542) and Anti‐phospho‐eNOS (Abcam, #ab184154) were commercially purchased from companies. Anti‐Phospho‐AKT (#4060), Anti‐AKT (#9272) and Anti‐eNOS (#32027) were purchased from Cell Signaling Technology. Anti‐S1pr1 antibody (PA1‐1040) was purchased from ThermoFisher. Total Nitric Oxide Assay Kit was purchased from Beyotime (Beyotime, #S0024). Mouse cGMP ELISA was purchased from Shanghai enzyme linked Biotechnology (#ml001887‐1). NS‐2028 was purchased from Beyotime (#S1769).

### 2.5 Histology

The hearts were harvested and then fixed with 4% paraformaldehyde overnight. Serial sections were obtained at 6 μm intervals for paraffin‐embedded tissue. We stained serial sections with wheat germ agglutinin (WGA) for analysis of myocyte cross‐sectional areas. Biotinylated‐isolectin B4 (IB4) staining was used for cardiac microvessel density measurement. Masson's trichrome staining was applied for measurement of cardiac fibrosis. Images were collected by a Leica microscope (DM6000B; Leica). Immunofluorescence staining was performed with primary antibody and the corresponding secondary antibodies, including Alexa Fluor 488‐conjugated donkey antimouse secondary antibodies (2 µg/mL, A21206; Invitrogen) and Alexa Fluor 594‐conjugated donkey antirabbit secondary antibodies (2 µg/mL; Invitrogen). Slides were mounted with Vectashield mounting medium containing DAPI.

### 2.6 Cell culture and supernatant collection

HUVECs were cultured in Dulbecco's modified Eagle's medium (DMEM) supplemented with 10% foetal bovine serum (FBS), 100 U/mL penicillin and 100 μg/mL streptomycin. The cells were seeded at a density of 1 × 10^6^ per well into 6‐well culture plates and at a density of 3.0 × 10^3^ per well in 96‐well plates; cells at passage 3‐6 were used for experiments. After serum‐starvation for 12 hours, HUVECs were treated with VEGF (50 ng/mL) in the presence or absence of L‐NAME (100 μmol/L) or LY294002 (25 μmol/L) or their vehicles for 24 hours, and then HUVEC‐conditioned supernatant were collected for further experiments.

### 2.7 Isolation and culture of primary rat cardiomyocytes

Rat neonatal cardiomyocytes were isolated as reported previously.^3^ In brief, neonatal cardiomyocytes were obtained from 1‐day‐old neonatal rat hearts by trypsinization and collagenization and hatched at 37°C in humidified air containing 5% CO_2_. The single cell suspensions were plated on 100‐mm culture dishes in DMEM with 10% foetal bovine serum for 2 hours. The non‐attached cardiomyocyte fraction was counted and plated on plastic dishes with 100 μmol/L bromodeoxyuridine to restrain fibroblast proliferation. Before neonatal cardiomyocytes co‐cultured with HUVEC‐conditioned medium, cardiomyocytes were starved for 12 hours. Cardiomyocytes were then treated with 500 nmol/L AngII for additional 48 hours to induce cell hypertrophy in vitro.

### 2.8 Isolation of ECs and SMCs

Mice were anaesthetized and sacrificed. The mouse hearts were excised aseptically and minced finely, followed by incubation in PBS buffer containing 1 mg/mL collagenase I (Sigma) and 60 units/mL DNase I at 37°C for 60 minutes with continuous and gentle agitation. The cell suspension was then passed through a 70 μm cell strainer and centrifuged. The cell pellet was resuspended in 0.1% bovine serum albumin (BSA)/PBS with anti‐CD31 antibody‐conjugated Dynabeads (Invitrogen). The cell suspension rotated for 20 minutes and then subjected to magnetic separator. After separation in magnetic separator, the isolated ECs were plated on gelatin‐coated dishes. ECs were cultured in endothelial cell culture medium containing 10% FBS and 30 μg/mL ECGS (Corning). When the density of ECs was 80%‐90% confluency, they were purified again by anti ICAM‐2 antibody‐conjugated Dynabeads. The purified ECs were used for further experiments.

Eight‐ to twelve‐week‐old mice were sacrificed and their aortas were excised from the arch to the iliac bifurcation. The media of aorta was dissected under microscope and cut into 4 mm long pieces then covered by an autoclaved glass coverslip. Smooth muscle cells were cultured in Dulbecco's modified Eagle's medium supplemented with 20% foetal bovine serum at 37°C, 5% CO_2_ and used for further experiments.

### 2.9 Measurement of nitric oxide concentration

Total NO production in culture medium was determined by measuring the concentration of nitrate and nitrite, a stable metabolite of NO, by modified Griess reaction method. The NO content in each group was detected and calculated according to the instructions of the Total Nitric Oxide Assay Kit.

### 2.10 Measurement of mouse cyclic guanosine monophosphate concentration

Cells were lysed by repetitive freeze‐thaw cycles and cell lysates were centrifuged at the speed of 14 000 *g* for 15 minutes at 4°C. The concentration of cyclic guanosine monophosphate (cGMP) in fibroblast lysates was measured and calculated according to the protocol of Mouse cGMP ELISA Kit (#ml001887‐1, Shanghai enzyme linked Biotechnology).

### 2.11 Measurement of cell surface area

The rat cardiomyocytes were seeded at a density of 2.0 × 10^4^ per well in 24‐well plates on sterile glass coverslips and then fixed and stained with TRITC‐phalloidin (Yeasen, #40734ES75) at a dilution of 1:80 in phosphate‐buffered saline (PBS). After washing with PBS, the cells were incubated with Vectashield mounting medium containing DAPI. A single cell area was measured with ImageJ software. At least 50 cells per group were measured in each experiment.

### 2.12 Quantitative real‐time PCR

Total RNA was extracted by Trizol and cDNA synthesized by SuperScript First Strand Synthesis System (Takara). RT‐qPCR was performed with PowerUp SYBR Green master mix (Applied Biosystems, #A25741) on a QuantStudio 6 Flex Real‐time PCR system (Applied Biosystems) according to the manufacturer's protocol. Transcript quantities were normalized to *gapdh* expression levels. Primer sequences are given in Table 1.

**Table 1:** Primer sequence for real‐time quantitative PCR

Gene name	Primer sequence
*R‐anf*	Forward: 5′‐*ATACAGTGCGGTGTCCAACA‐*3′
	Reverse: 5′‐*AGCCCTCAGTTTGCTTTTCA*‐3′
*R‐bnp*	Forward: 5′‐*TTGGGCAGAAGATAGACCGGAT*‐3′
	Reverse: 5′‐*GGTCTTCCTAAAACAACCTCA‐*3′
*R‐myh7*	Forward: 5′‐*AACCTGTCCAAGTTCCGCAAGGTG*‐3′
	Reverse: 5′‐*GAGCTGGGTAGCACAAGAGCTACT*‐3′
*R‐gapdh*	Forward: 5′‐*TTGCCATCAACGACCCCTTC*‐3′
	Reverse: 5′‐*TTGTCATGGATGACCTTGGC*‐3′
*m‐alpha‐sma*	Forward: 5′‐*GTCCCAGACATCAGGGAGTAA*‐3′
	Reverse: 5′‐*TCGGATACTTCAGCGTCAGGA*‐3′
*m‐col3*	Forward: 5′‐*CTGTAACATGGAAACTGGGGAAA*‐3′
	Reverse: 5′‐*CCATAGCTGAACTGAAAACCACC*‐3′
*m‐gapdh*	Forward: 5′‐*AGGTCGGTGTGAACGGATTTG*‐3′
	Reverse: 5′‐*GGGGTCGTTGATGGCAACA*‐3′

### 2.13 Western blotting

Cells in each group were washed with pre‐cooled PBS three times and lysed in RIPA lysis buffer (Beyotime, #P0013C). The product was centrifuged at 14 000 *g* for 10 minutes at 4°C. The supernatant was obtained and protein concentrations were determined by a BCA kit (Beyotime, #P0010). Thirty microgram of total proteins were separated by 10% SDS‐PAGE, transferred to PVDF membranes (Millipore). The membranes were soaked in 5% skimmed milk for 2 hours and incubated overnight at 4°C with the corresponding primary antibodies. After incubation with the corresponding secondary antibodies at room temperature for 2 hours, the immune complexes were visualized by the Odyssey infrared imaging system (Li‐Cor).

### 2.14 Statistical analysis

Data were presented as means ± SEM unless otherwise noted. Shapiro‐Wilk test was used to determine the normality of data and all data in the article passed the normality distribution test. Comparisons between two groups were analysed by 2‐tailed Student's *t* test*.* Differences between multiple groups were performed with one‐way ANOVA, followed by the Turkey post hoc tests. *P* < .05 was considered to denote statistical significance. All statistical analyses were performed by SPSS 20.0 software package (SPSS Inc.).

## 3 RESULTS

### 3.1 S1pr1 expression is up‐regulated in ECs after TAC and loss of EC‐S1pr1 increases cardiac mass and worsens cardiac dysfunction induced by TAC

**Figure 1:**
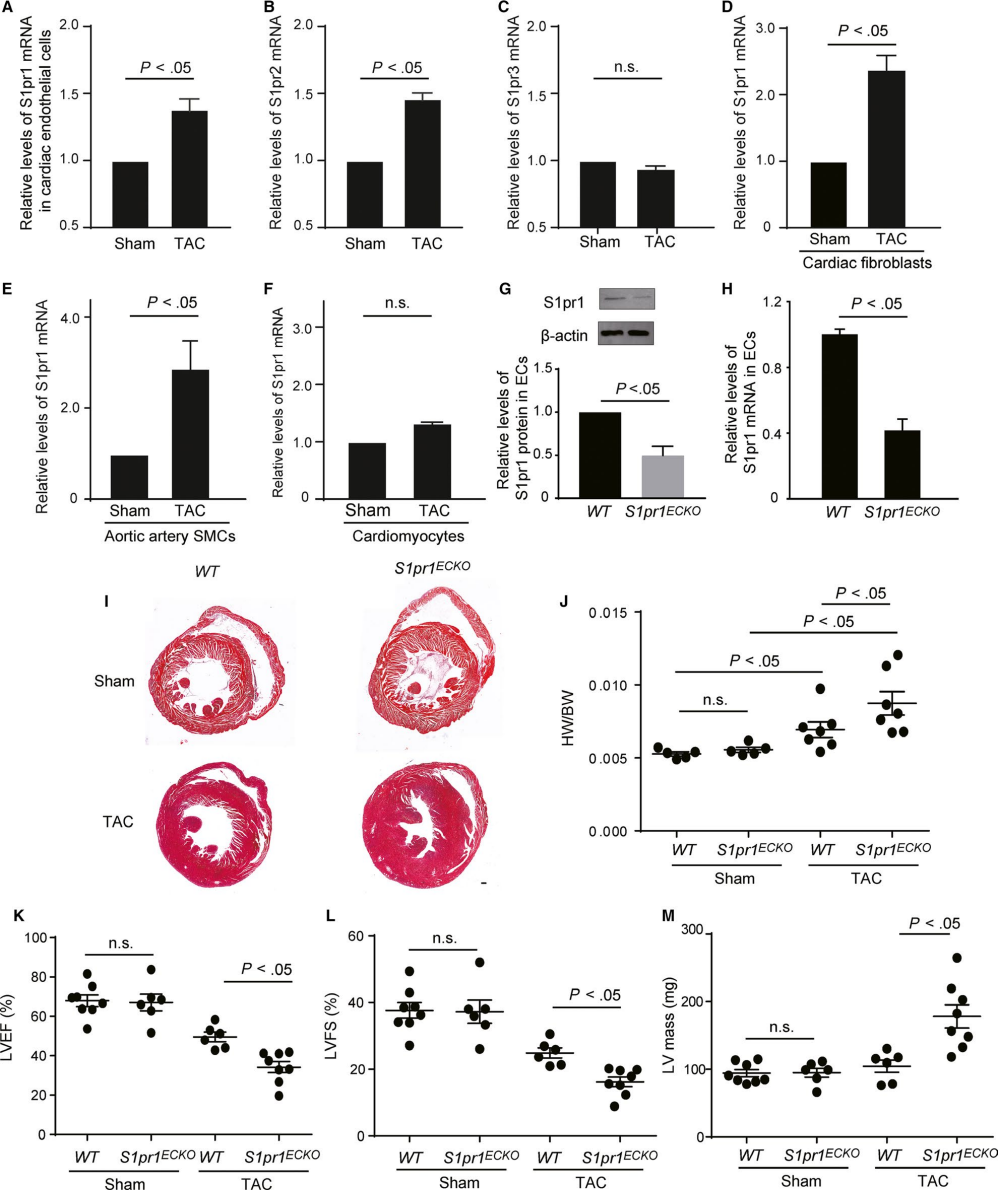
Loss of endothelial S1pr1 worsens cardiac dysfunction after transverse aortic constriction (TAC) operation. A‐C, Relative mRNA expression levels of S1pr1 (A), S1pr2 (B) and S1pr3 (C) in cardiac endothelial cells obtained from mice with sham or TAC operation. n = 3. D‐F, Relative mRNA expression levels of S1pr1 in cardiac fibroblasts (D), aortic artery smooth muscle cells (SMCs) (E) and cardiomyocytes (F) obtained from mice with sham or TAC operation. n = 3. G, Western blotting analysis of S1pr1 protein levels in ECs from EC conditional *S1pr1* knock‐out mice or WT control mice, with their quantification. n = 3. H, Relative mRNA expression levels of S1pr1 in ECs of *WT* and *S1pr1^ECKO^* mice. n = 3. I, Representative images of H&E staining of TAC hearts in *WT* and *S1pr1^ECKO^* mice with sham or TAC operation. n = 5‐6. J, Quantification of the ratio of heart weight (HW) to bodyweight (BW) in *WT* and *S1pr1^ECKO^* mice after sham or TAC operation. n = 5‐7. K‐L, Quantification of left ventricle (LV) ejection fraction (EF%) (K), LV fractional shortening (LVFS%) (L) and LV mass (M) measured by echocardiography. n = 6‐8. Scale bar, 100 μm. Data are mean ± SEM

To induce pathological cardiac remodelling, we performed transverse aortic constriction (TAC) operation in mice to establish a chronic pressure overload‐induced heart failure. We first measured the expression levels of S1pr1 in hearts following TAC in mice. Our data showed that S1pr1 was not altered in the banded hearts (data not shown). We further measured the expression levels of S1pr1 in ECs of the banded hearts. Our results showed that S1pr1 expression was significantly up‐regulated in cardiac ECs of hearts following TAC operation, in comparison with the sham group (Figure 1A). Besides S1pr1, our data showed that the expression levels of S1pr2 were up‐regulated in ECs of the TAC hearts, while S1pr3 was not altered in cardiac ECs by TAC operation (Figure 1B,C). We also measured the expression levels of S1pr1 in cardiac fibroblasts (CFs), aortic artery smooth muscle cells (SMCs) and cardiomyocytes (CMs) after transverse aortic constriction. Our results showed that S1pr1 levels were up‐regulated in CFs and SMCs, not in CMs (Figure 1D‐F). Considering that S1pr1 was highly expressed in endothelial cells and that cardiac vascular endothelial cells were the major cell type in the hearts, the above data suggest that endothelial cell‐expressing S1pr1 might be involved in the pathological process of pressure overload‐induced heart failure. To address our hypothesis, we established drug inducible endothelial cell‐specific deletion of *S1pr1* mice by crossing *Cdh5‐Cre^ERT^* mice with *S1pr1^flox/flox^* mice, simplified as *S1pr1^ECKO^* (Figure 1G). Following the induction of Cre recombinase activity by treatment with tamoxifen (100 mg/kg for four times), S1pr1 expression was specifically reduced in the ECs of *S1pr1^ECKO^* mice, as shown by Western blotting and quantitative RT‐PCR (Figure 1G,H).

After TAC operation, alterations of bodyweight were not observed between groups (data not shown); however, the ratio of heart‐to‐body weight was significantly increased in *S1pr1^ECKO^* mutants, compared to *WT* control mice (Figure 1I,J). In consistence with this, echocardiographic measurements showed a remarkable increase in LV mass of *S1pr1^ECKO^* (Figure 1M). Furthermore, a significant reduction in cardiac systolic function was observed in *S1pr1^ECKO^* groups, as shown by left ventricular ejection fraction (EF) (Figure 1K) and fractional shortening (FS) (Figure 1L).

Taken together, S1pr1 is highly expressed in cardiac ECs and up‐regulated during the process of pressure overload‐induced heart failure. Specific EC‐S1pr1 deletion in mice aggravates cardiac hypertrophy and deteriorates cardiac function during chronic afterload‐induced heart failure.

### 3.2 Loss of EC‐S1pr1 promotes pressure overload‐induced cardiac fibrosis through enhancing cardiac fibroblast proliferation and myofibroblast formation in vivo

**Figure 2:**
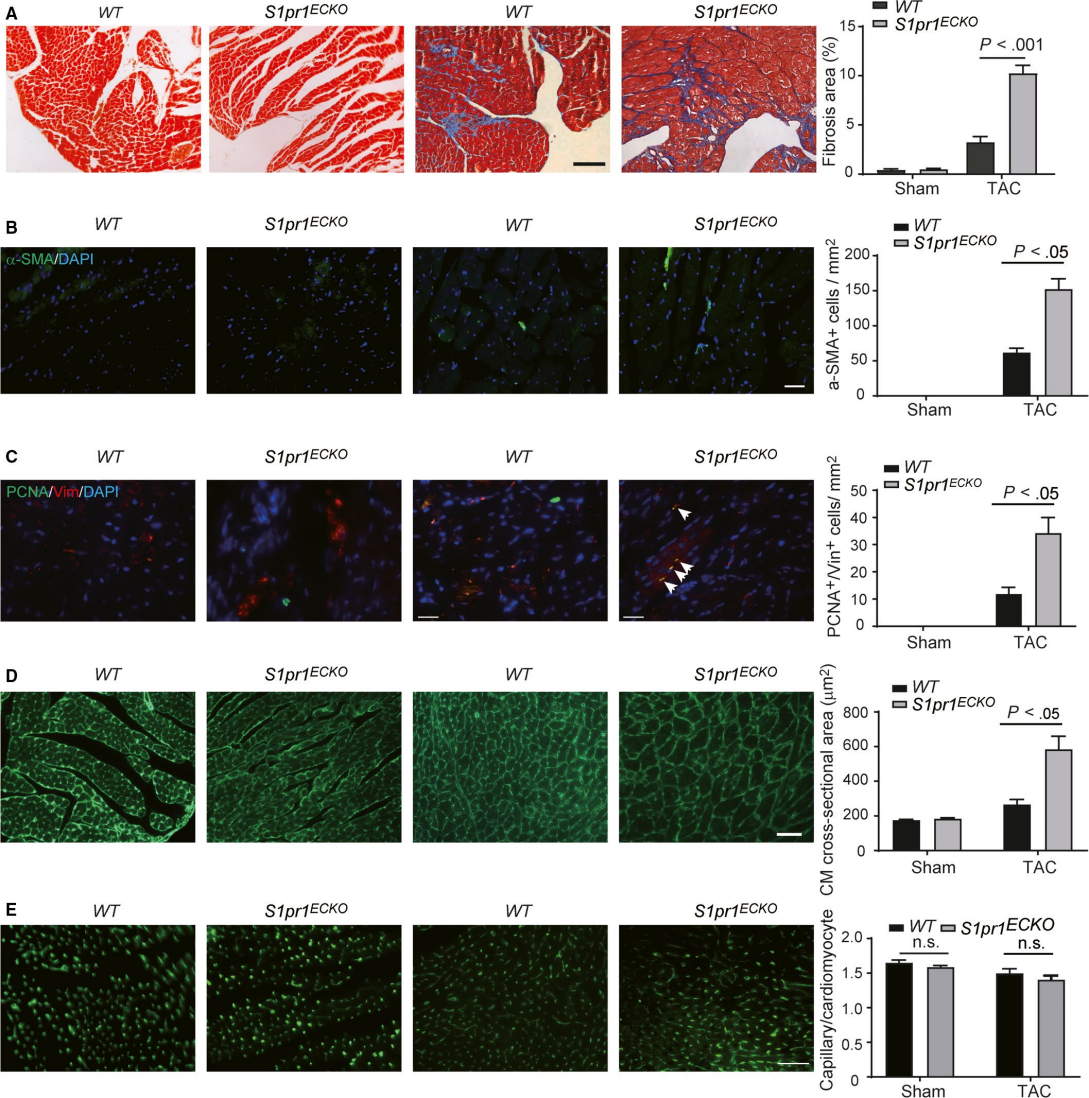
Endothelial S1pr1‐deficiency aggravates post‐TAC pathological cardiac remodelling. A, Representative images of Masson's Trichrome staining of sham or TAC hearts in *WT* and *S1pr1^ECKO^* mice, with quantification of the percentage of cardiac fibrosis in left ventricle myocardium. n = 6. B, Representative images of immunostaining of α‐SMA in the sham or TAC hearts of *WT* and *S1pr1^ECKO^* mice, with their quantification. n = 6. C, Representative images of co‐immunostaining of PCNA/Vimentin (Vim) in sham or TAC hearts of *WT* and *S1pr1^ECKO^* mice, with quantification of PCNA^+^ cells in Vimentin (Vim)^+^ fibroblasts. Arrows indicate PCNA^+^Vim^+^ fibroblasts. n = 6. D, Representative images of WGA staining of sham or TAC hearts in *WT* and *S1pr1^ECKO^* mice, with quantification of the cross‐sectional cardiomyocyte area in hearts. n = 6. E, Representative images of isolectin‐B4 staining of TAC hearts in *WT* and *S1pr1^ECKO^* mice, with quantification of capillary density in myocardium after sham or TAC operation. n = 6. Data are mean ± SEM. Scale Bars: A, 100 μm; C, 25 μm; B, D and E, 50 μm. n.s., no statistic significance

We next examined whether EC‐S1pr1 was involved in the regulation of cardiac fibrosis after TAC operation. Our data showed that loss of EC‐S1pr1 resulted in a greater cardiac fibrosis in TAC hearts, as shown by Masson's Trichrome staining (Figure 2A). We further observed more myofibroblast formation in post‐TAC hearts of *S1pr1^ECKO^* mice, compared to *WT* littermates, as shown by immunostaining of myofibroblast marker, α‐SMA (Figure 2B). Since fibroblast proliferation significantly influences myofibroblast population during cardiac remodelling, we next investigated the proliferation capability of fibroblast by immunostaining of proliferating cell nuclear antigen (PCNA) and fibroblast marker, vimentin, in TAC hearts. We observed that loss of EC‐S1pr1 led to more proliferating fibroblasts during pressure overload‐induced cardiac fibrosis, compared with wild‐type littermates (Figure 2C). Taken together, our data suggested that EC‐S1pr1 regulated pathological cardiac fibrosis via increasing fibroblast proliferation and promoting myofibroblast formation.

### 3.3 Endothelial S1pr1 deficiency aggravates cardiac hypertrophy but not influence capillary density in hearts after TAC operation

We next performed histological analysis to investigate the role of EC‐S1pr1 for cardiac remodelling induced by TAC. Immunostaining of Wheat Germ Agglutinin (WGA), which binds to glycoprotein of the cell membrane, showed that loss of EC‐S1pr1 significantly increased cardiomyocyte cross‐sectional area in the mice following TAC operation (Figure 2D), while cardiac microvessel density was not altered in *S1pr1^ECKO^* mice as shown by isolectin‐B4 staining in banded hearts (Figure 2E). These results suggested that EC‐S1pr1 was deeply involved in the process of pressure overload‐induced pathological cardiac hypertrophy.

### 3.4 Loss of endothelial S1pr1 enhances fibroblast proliferation, migration and its conversion towards myofibroblast with more extracellular matrix component production via AKT/eNOS pathway

**Figure 3:**
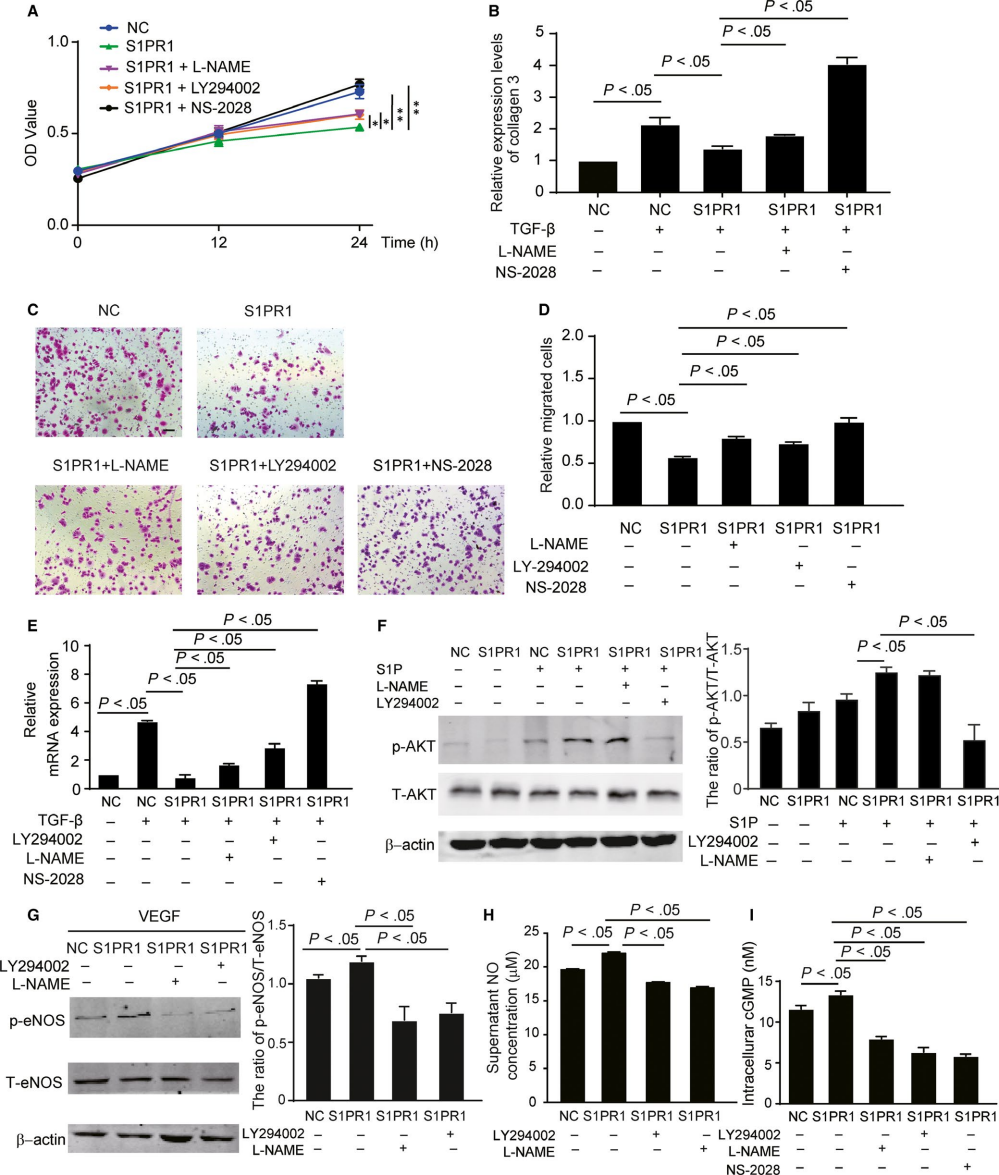
Endothelial S1pr1 inhibits fibroblast proliferation, migration and its conversion towards myofibroblasts via AKT/eNOS pathway. A, Condition medium of lentiviral S1PR1‐overexpressing ECs decreases fibroblast cell proliferation, while blockade of AKT or eNOS in ECs and inhibition of sGC in fibroblasts increase cell proliferation, as shown by MTT assay. n = 5. B, Relative expression levels of collagen 3 in fibroblasts treated with HUVEC‐conditioned medium in the indicated groups. n = 5. C, D, Condition medium of lentiviral S1PR1‐overexpressing ECs reduced MCF cell migration, while blockade of AKT or eNOS in ECs and inhibition of sGC in fibroblasts enhance cell migration, as shown by transwell assay. HPF, high‐power field. n = 3. E, Relative expression levels of α‐SMA in fibroblasts treated with HUVEC‐conditioned medium in the indicated groups. n = 5. F, Western blotting of AKT activation status in HUVECs treated with or without S1P in the indicated groups with quantification. n = 5. G, Western blotting of eNOS activation status in HUVECs treated with or without S1P in the indicated groups with quantification. n = 5. H, Nitric oxide (NO) production in HUVEC‐conditioned medium in the indicated groups. n = 5. I, cGMP levels in fibroblasts of the indicated groups. n = 5. NC, scramble shRNA lentivirus. S1PR1, S1PR1‐overexpressing lentivirus. shRNA, S1PR1 shRNA lentivirus. L‐NAME, eNOS antagonist. LY294002 (LY), AKT inhibitor. NS‐2028, sGC inhibitor. Scale bar, 100 μm. Data are mean ± SEM. *P < .05. **P < .01

To investigate more details in the effect of EC‐S1pr1 on fibroblast proliferation, migration and its conversion towards myofibroblast, we co‐cultured fibroblasts with the conditioned medium obtained from S1PR1‐overexpressing ECs. Overexpression of S1PR1 significantly inhibited fibroblast proliferation shown by MTT assay (Figure 3A). As expected, S1PR1‐overexpressing EC‐conditioned medium reduced extracellular matrix component, collagen 3, production by fibroblast (Figure 3B). We further examined whether EC‐S1pr1 controlled fibroblast migration in a paracrine manner, our transwell assay showed that S1PR1‐overexpressing EC‐conditioned medium decreased fibroblast migration (Figure 3C,D). We next detected lower expression of α–SMA (myofibroblast marker) in fibroblasts co‐cultured with S1PR1‐overexpressing EC‐conditioned medium, which was consistent with our in vivo observations that EC‐S1pr1 influenced myofibroblast formation (Figure 3E). These results confirmed that EC‐S1pr1 deficiency promoted fibroblast proliferation, migration and its conversion of fibroblasts towards myofibroblasts to produce more extracellular matrix components in a paracrine manner.

It has been shown that S1P activated AKT signalling pathway via S1pr1/Gi in various cell types.^10^ To test whether AKT activity could be enhanced by S1P/S1pr1 stimulation in endothelial cells, we performed Western blot analysis and our results showed that the active levels of AKT was significantly higher in S1PR1‐overexpressing HUVECs (human umbilical venous endothelial cells) after S1P challenge (Figure 3F), suggesting S1P activated AKT signalling pathway via S1pr1 in ECs. In endothelial cells, eNOS signalling pathway plays an essential role in the regulation of endothelial function and has been shown as one downstream target of AKT signalling pathway.^11^, ^12^ We next investigated whether S1P/S1PR1 praxis influenced eNOS signalling pathway in ECs. Our Western blot analysis showed that S1P increased the levels of phospho‐eNOS in S1PR1‐overexpressing HUVEC triggered by VEGF (50 ng/mL), and this phenotype was inhibited by eNOS antagonist, L‐NAME (Figure 3G). Moreover, inhibition of AKT signalling pathway by AKT inhibitor, LY294002, decreased the enhanced eNOS activity in S1PR1‐overexpressing HUVECS (Figure 3G), suggesting that S1P/S1PR1 praxis activates eNOS via AKT signalling pathway. As expected, S1PR1‐expressing HUVECs produced and secreted more NO into the cell culture supernatant, while inhibition of AKT/eNOS reduced NO production in ECs (Figure 3H). To further investigate whether the effects of EC‐S1pr1 on fibroblast is dependent on AKT/eNOS signalling pathway, we treated fibroblasts with AKT/eNOS inhibitors in the presence of the conditioned medium obtained from S1PR1‐overexpressing ECs in vitro. Both AKT inhibitor, LY294002, and eNOS antagonist, L‐NAME, reversed the inhibitory effects of EC‐S1pr1 on fibroblast proliferation, migration and its conversion towards myofibroblast in vitro (Figure 3A‐E).

It is known that endothelium‐derived NO binds to its receptor, soluble guanylate cyclase (sGC) to stimulate the synthesis of cGMP in fibroblast.^13^ We next examined whether cGMP levels in fibroblast was influenced by EC‐expressing S1PR1 via NO/sGC pathway. Our data showed that conditioned medium obtained from S1PR1‐overexpressing ECs significantly up‐regulated cGMP levels in fibroblasts, while blockade of AKT/eNOS or inhibition of sGC in fibroblasts reduced cGMP levels (Figure 3I). Previous investigations have reported that cGMP reduced fibroblast proliferation, fibroblast‐to‐myofibroblast differentiation, and extracellular matrix production.^13^ We next investigated whether the effect of EC‐expressing S1PR1 on fibroblasts via NO/sGC/cGMP signalling pathway. As expected, inhibition of sGC/cGMP signalling pathway reversed the inhibitory effect of EC‐S1pr1 on fibroblast proliferation, migration and its conversion towards myofibroblast in vitro (Figure 3A‐E). Taken together, these results suggest that EC‐S1pr1 might control pathological cardiac remodelling via AKT/eNOS/NO/sGC/cGMP pathway.

### 3.5 Endothelial S1pr1reduced cardiomyocyte hypertrophy via AKT/eNOS pathway

**Figure 4:**
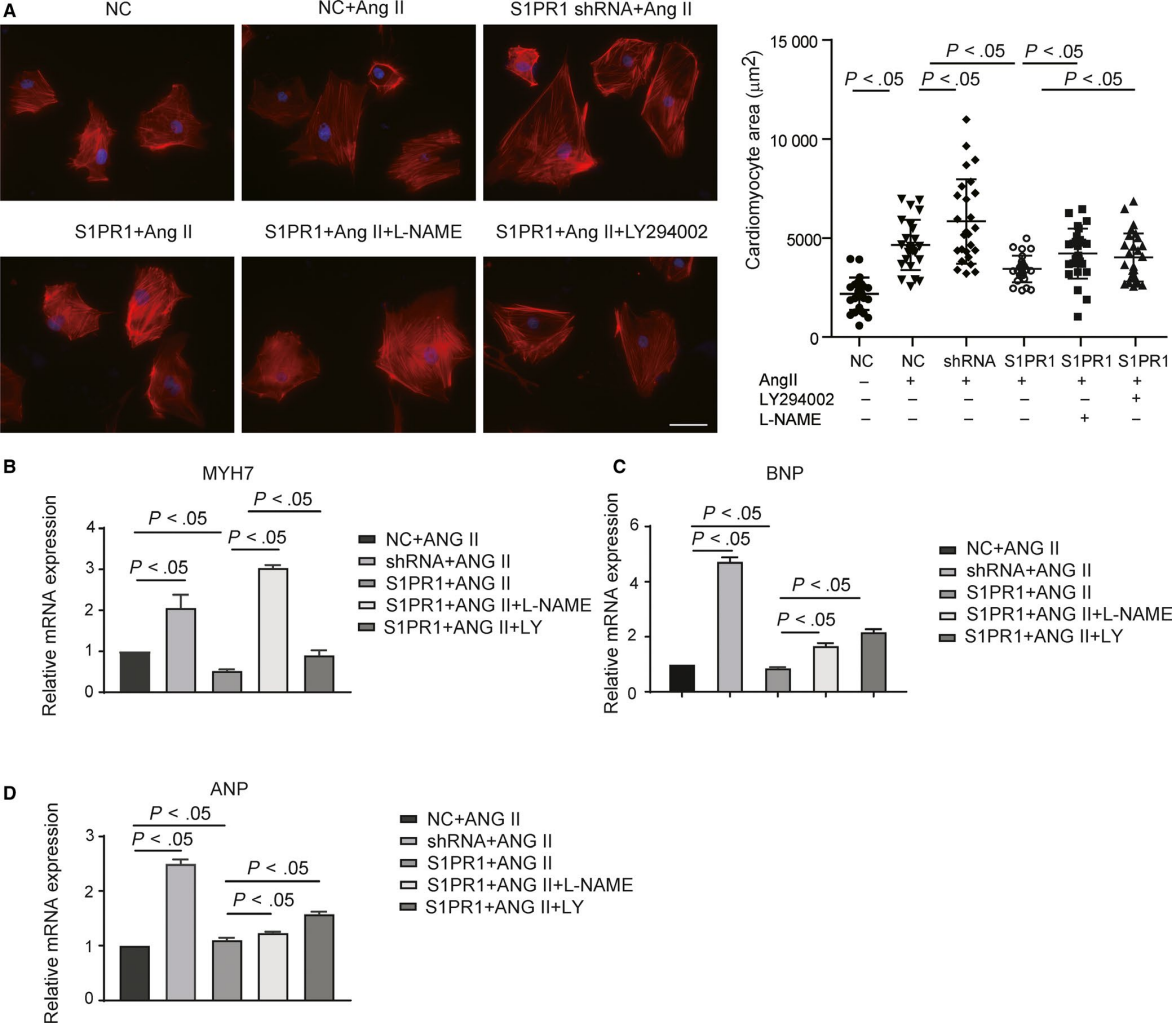
Endothelial S1pr1 reduced cardiomyocyte hypertrophy via AKT/eNOS pathway. A, Condition medium of lentiviral S1PR1‐overexpressing ECs decreases CM area, while S1PR1 shRNA knockdown increases CM area, as shown by immunostaining of filament actin in rat neonatal cardiomyocytes (left), with quantification of cardiomyocyte size (right). Ang II, angiotensin II. n = 5. B‐D, Condition medium of lentiviral S1PR1‐overexpressing ECs decreases MYH7 (B), BNP (C) and ANP (D) expression, while S1PR1 shRNA knockdown increases MYH7 (B), BNP (C) and ANP (D) expression, as shown by RT‐PCR. n = 5. NC, scramble shRNA lentivirus. S1PR1, S1PR1‐overexpressing lentivirus. shRNA, S1PR1 shRNA lentivirus. L‐NAME, eNOS antagonist. LY294002 (LY), AKT inhibitor. Data are mean ± SEM. Scale Bars: A, 50 µm

It has been shown that eNOS/NO signalling pathway played a protective role in the pathogenesis of cardiac hypertrophy.^14^ We hypothesized that endothelial S1pr1 might regulate cardiac hypertrophy by activating AKT/eNOS signalling pathway. Many studies have shown that renin‐angiotensin system (RAS) was activated in response to pressure overload in TAC animal model, and that angiotensin II (AngII) significantly contributed to the cardiomyocyte hypertrophy. We therefore treated cardiomyocyte with AngII in vitro to induce cell hypertrophy and examined whether EC‐S1pr1 influenced CM hypertrophy in a paracrine manner.^15^ In vitro co‐culture experiments showed that the conditioned medium obtained from S1PR1‐overexpressing ECs reduced cardiomyocyte hypertrophy, while the blockade of AKT/eNOS signalling diminished this inhibitory effect of EC‐S1pr1 on cardiac hypertrophy (Figure 4A). It was well known that the foetal gene program was reactivated in the pathophysiological process of cardiac hypertrophy,^3^ we next investigated the influences of EC‐S1pr1 on the expression of foetal genes in cardiomyocytes. Our studies showed that the markers for the foetal gene program, including ANP, BNP and MYH7, were significantly down‐regulated in the cardiomyocytes treated with the conditioned medium obtained from S1pr1‐overexpressing ECs, compared with the medium from control ECs (Figure 4B‐D). As expected, the inhibition of AKT/eNOS signalling pathway increased the expression of ANP, BNP and MYH7 in cardiomyocytes treated with S1pr1‐overexpresssing EC medium (Figure 4B‐D), suggesting that EC‐S1pr1 inhibited the pathophysiological process of cardiac hypertrophy via AKT/eNOS signalling pathway.

### 3.6 Pharmacological activation of S1pr1 attenuates pressure overload‐induced cardiac fibrosis and hypertrophy, and ameliorates the cardiac dysfunction

**Figure 5:**
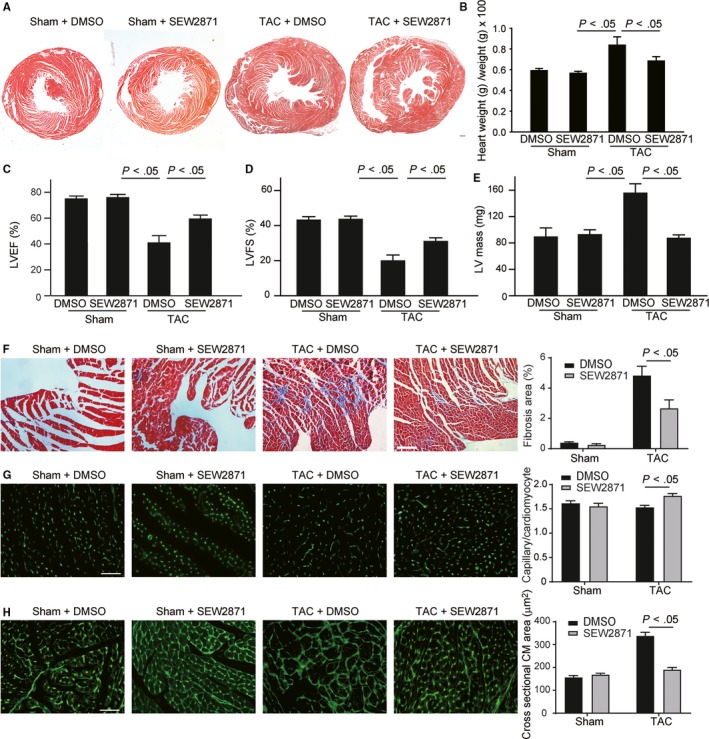
Pharmacological activation of S1pr1 ameliorates cardiac dysfunction and remodelling after transverse aortic constriction (TAC) operation. A, Representative images of H&E staining of sham or TAC hearts treated with or without S1pr1 agonist, SEW2871. n = 5‐7. B, Quantification of the ratio of heart weight to bodyweight in the indicated groups. n = 5‐7. C‐E, Quantification of left ventricle (LV) ejection fraction (LVEF%) (C), Left ventricle fractional shortening (LVFS%) (D) and LV mass (E) measured by echocardiography. n = 5‐7. F, Representative images of Masson's Trichrome staining of sham or TAC hearts in mice treated with or without SEW2871, with quantification of the percentage of cardiac fibrosis in left ventricle myocardium. n = 5‐7. G. Representative images of isolectin‐B4 staining of hearts in the indicated groups with quantification of capillary density in myocardium after sham or TAC operation. n = 5‐7. H, Representative images of WGA staining of sham or TAC hearts in the indicated groups, with quantification of the cross‐sectional cardiomyocyte area in hearts. n = 5‐7. Data are mean ± SEM Scale Bars: A, F, 100 μm. G‐H, 50 μm

**Figure 6:**
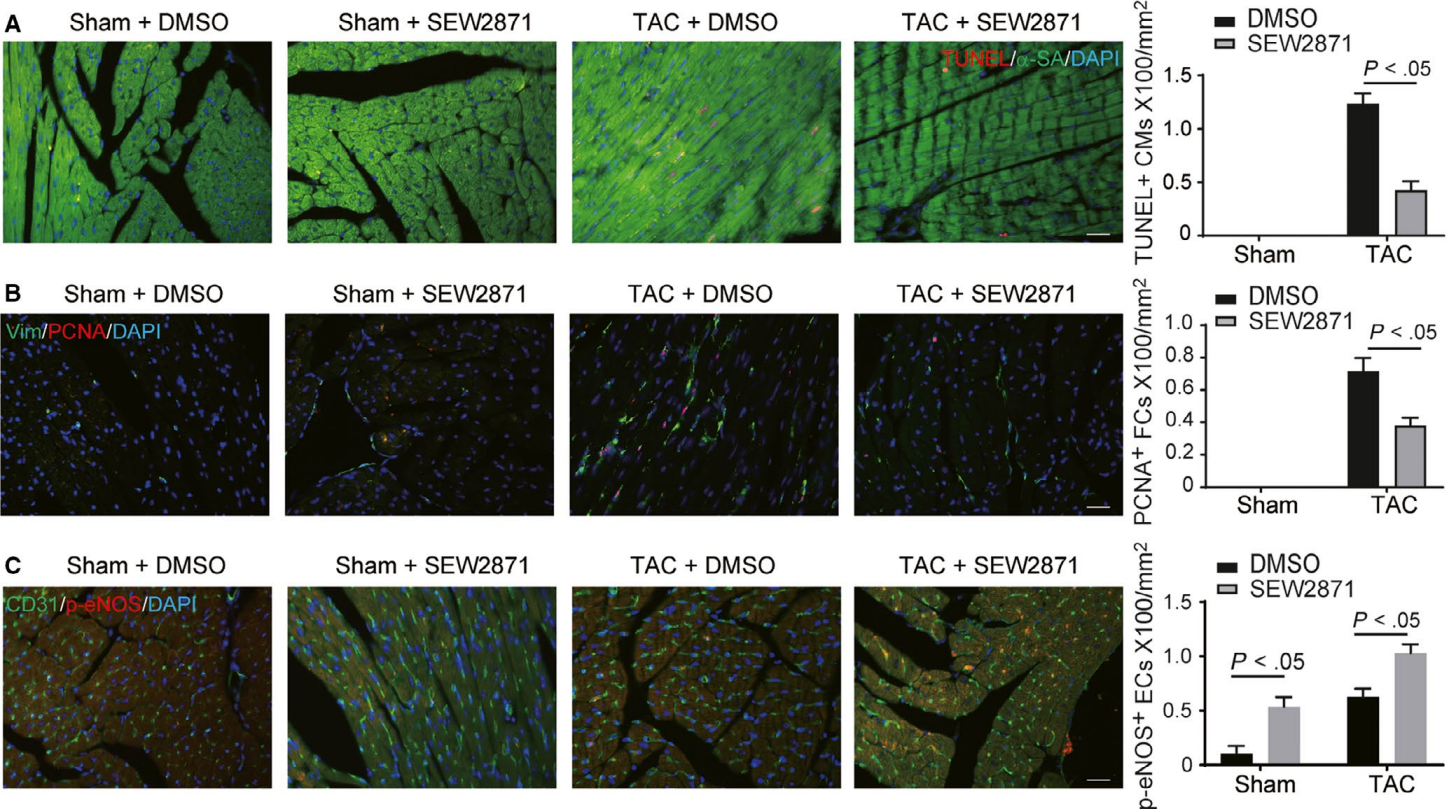
Pharmacological activation of S1pr1 reduced cell apoptosis of cardiomyocytes and enhanced eNOS activity in ECs and reduced cardiac fibroblast proliferation during pressure overload‐induced cardiac remodelling. A, Representative images of TUNEL staining of hearts in the indicated groups with quantification of TUNEL^+^ cardiomyocytes in myocardium after sham or TAC operation. n = 5. B, Representative images of PCNA staining of hearts in the indicated groups with quantification of PCNA^+^ fibroblast in myocardium after sham or TAC operation. n = 5. C, Representative images of p‐eNOS staining of cardiac ECs in the indicated groups with quantification of p‐eNOS^+^ ECs in myocardium after sham or TAC operation. n = 5. Data are mean ± SEM. Scale Bars: A‐C, 50 μm

Since genetic EC‐S1pr1 overexpression in vitro can inhibit cardiomyocyte hypertrophy and myofibroblast accumulation, we next tested whether pharmacological activation of S1pr1 has a beneficial effect on pathological cardiac remodelling during chronic heart failure. Pharmacological activation of S1pr1 by S1pr1 agonist, SEW2871 (5 mg/kg/d) for 4 weeks significantly reduced cardiac hypertrophy, as shown by the ratio of HW (heart weight)/BW (bodyweight) (Figure 5A,B) and LV mass measured by echocardiography (Figure 5E). Activation of S1pr1 signalling significantly increased LVEF% and LVFS% in TAC model (Figure 5C,D), indicating that SEW2871 improves cardiac function during the development of heart failure. Our further histological study showed that SEW2871 reduced TAC‐mediated cardiac fibrosis, compared to the control group (Figure 5F). Furthermore, SEW2871 agonist increased capillary density and diminished cardiomyocyte size in banded hearts (Figure 5G,H), suggesting S1pr1 activation improved cardiac remodelling during pressure overload‐induced heart failure. It has been showed that SEW2871 decreased cardiomyocyte apoptosis and improved cell survival after heart injury.^16^ Therefore, we next examined cardiomyocyte apoptosis after TAC operation by TUNEL staining. As expected, our data showed that activation of S1pr1 reduced cardiomyocyte apoptosis after TAC operation (Figure 6A). We further detected less cardiac fibroblast proliferation in post‐TAC hearts treated with SEW2871, as shown by immunostaining of PCNA (Figure 6B). To examine whether eNOS signalling pathway was activated by SEW2871, we performed immunostaining of phospho‐eNOS in TAC hearts. In consistence with our in vitro results, SEW2871 treatment enhanced eNOS activity of cardiac ECs in TAC hearts (Figure 6C). Taken together, our results suggest that activation of S1pr1 could ameliorate cardiac remodelling during chronic heart failure and therefore provide SEW2871 as a possible novel therapeutic target against heart failure.

## 4 DISCUSSION

Vascular endothelial cells are the major cell type in the heart and their homeostasis plays an essential role in cardiac remodelling during the development of heart failure. The interplay of cardiac endothelial cells with their adjacent cardiomyocytes, fibroblast and other cells within myocardium tightly controls the pathological process of cardiac remodelling, including cardiac hypertrophy and cardiac fibrosis.^3^, ^17^ Besides its contribution to vascularization, cardiac endothelium secretes multiple factors, for example NO, to influence other cardiac cells in a paracrine manner.

Endothelial cells‐derived NO has been shown a protective effect on heart after injury.^18^ Stretch or shear stress activates eNOS synthase to produce and secrete NO, resulting in vasodilation of smooth muscle cells and protecting from hypertrophy and fibrosis.^18^, ^19^ Pharmacological activation of eNOS attenuated hypertension‐induced diastolic dysfunction.^20^ Other studies showed that eNOS‐deficiency exacerbates adverse cardiac remodelling when mice are exposed to pressure overload induced by TAC.^21^ Previous studies indicated endothelial S1pr1 was involved in the regulation of eNOS signalling pathway via activation of Akt signalling.^22^ Our investigation demonstrated that endothelial S1pr1 was involved in the regulation of AKT/eNOS signalling pathway and improved the production of NO. A major finding of the present study is the significant aggravation of cardiac hypertrophy in mice lacking S1pr1 in endothelial cells after TAC operation. We demonstrated that one potential explanation may be due to impaired AKT/eNOS signalling in S1pr1‐deficient mice, as in vitro experiments showed that inhibition of AKT/eNOS reversed the inhibitory effect of S1pr1 on cardiac hypertrophy.

It has been shown that eNOS/NO/sGC/cGMP signalling pathway was involved in the regulation of cardiac fibroblast proliferation, migration and myofibroblasts differentiation, and therefore protected the heart from cardiac fibrosis during the pathological processes of chronic heart failure.^20^ As mentioned above, endothelial S1P/S1pr1 praxis activated AKT/eNOS signalling, suggesting that EC‐S1pr1 regulate cardiac fibrosis via its regulation of AKT/eNOS/NO/sGC/cGMP pathway. As expected, inhibition of AKT/eNOS or blockade of sGC in vitro reversed the inhibitory effect of S1pr1‐overexpressing EC‐conditioned medium on fibroblast migration, proliferation and myofibroblasts activation, explaining the molecular mechanism by which EC‐S1pr1 prevents heart failure development.

Previous investigations have showed a promising cardiac protective effect of S1P analogue.^23^ Fingolimod is a sphingosine 1‐phosphate receptor modulator that controls the egress of T lymphocytes from lymph nodes, and therefore prevents T lymphocytes from circulating to other tissues.^24^ Fingolimod has been approved to treat relapsing MS by the US Food and Drug Administration (FDA).^25^ In total, three Phase III studies demonstrated fingolimod reduced the annualized relapse rate (ARR) and improving MRI outcomes.^26^ Beside the therapeutic efficacy of fingolimod in MS, several reports pointed out fingolimod potential therapy for myocardial infarction. Wang et al^27^ reported that Fingolimod increases survival in adult murine cardiac myocytes subjected to hypoxia by inhibiting apoptosis and exhibits cardioprotective effects in vivo. Goltz et al^28^ have showed that Fingolimod displayed a better haemodynamics outcome in a mouse model of myocardial I/R. In particular, they reported that the beneficial effect of fingolimod on heart injury was associated with reduced phagocytic monocytes infiltrated the myocardium.^28^ More recently, Santos‐Gallego et al^29^ have reported that Fingolimod reduces cardiac fibrosis, and correspondingly improves cardiac function in pigs. Consistence with these investigations, our studies strongly supported that the overall notion that pharmacological activation of S1pr1 can improve cardiac remodelling and enhance cardiac functions in pressure overload‐induced heart failure. Our study further reveals an essential role of EC‐S1pr1 for the regulation of AKT/eNOS signalling pathway, which exerts cardiac protective effects and protects the injured heart from cardiac hypertrophy and cardiac fibrosis during chronic heart failure. This complements the molecular mechanism by which pharmacological activation of S1pr1 improves cardiac remodelling and enhances cardiac function in failing hearts. Although previous studies and our present report support a potential application of S1P analogue in heart diseases, we should take cautions on its side effect on heart rate.^30^ Some reports showed that fingolimod reduced heart rate (bradycardia) in rats.^30^ Gergely et al^31^ showed that an S1pr1 selective ligand results in transient bradycardia in humans. Therefore, other better drugs to improve S1P/S1pr1 signalling/function or therapeutic strategies to specific target cell‐specific S1P/S1pr1 signalling/function are currently under evaluation to avoid the side effect of S1pr1 modulator on cardiac conduction system.

In summary, our investigation showed an important role of EC‐S1pr1 in vivo pathological cardiac remodelling induced by pressure overload. Endothelial cells play an important role in the progression of pathological cardiac remodelling, and EC‐S1pr1‐AKT‐eNOS praxis restricts pathological cardiac fibrosis and hypertrophy improving cardiac function during heart failure development.

## CONFLICT OF INTEREST

None declared.

## AUTHOR CONTRIBUTIONS

L Zhang and Y Zhang conceived and designed the study; X Liu, J Wu and C Zhu performed experiments and acquired data; J Liu, X Chen, T Zhuang, Y Kuang, Y Wang and H Hu quantify experimental data; P Yu and H Fan revised the manuscript; Z Liu contributed intellectually and in the design of components of the studies and grant support. Overall, L Zhang and Y Zhang designed and oversaw the study and wrote the manuscript.

## Data Availability

The data that support the findings of this study are available from the corresponding author upon reasonable request.
